# A novel gyroscope based on the slow surface acoustic wave in a phononic metamaterial

**DOI:** 10.1038/s41378-024-00787-1

**Published:** 2024-11-14

**Authors:** Fei Ge, Liye Zhao, Jiawen Xu, Xukai Ding

**Affiliations:** Key Laboratory of Micro-Inertial Instrument and Advanced Navigation Technology, Ministry of Education, School of Instrument Science and Engineering, Southeast University, 210096 Nanjing, China

**Keywords:** Electrical and electronic engineering, Materials science, Physics

## Abstract

Limited to the direct modulation on the surface acoustic wave (SAW) by the rotation, the conventional SAW gyroscopes incur weak Coriolis effects and gyroscopic effects. In this paper, we innovatively utilize a phononic metamaterial (PM) operated at whispering-gallery modes (WGMs) as the vehicle for the Coriolis effect rather than SAW itself. The gyroscopic effects of this PM are investigated, and a new SAW gyroscope is subsequently proposed based on the slow SAW in PM. We show, combining theoretical modeling and finite element method simulation, that the rate of rotation can linearly induce the splitting of WGMs and modulate the phase velocity of SAW down to 4600 m/s (initial phase velocity of 5355 m/s); the direction of rotation results in the chiral symmetry of the PM vibration and the asymmetric distribution of the transmissive SAW. Besides, the proposed SAW gyroscope measures the angular velocity by detecting the phase shift resulting from rotation-dependent slow SAW in PM, obtaining a sensitivity of 0.016 deg/Hz when 50-cell PM. Compared with the existing SAW gyroscopes based on phase velocity modulation, the gyroscopic gain factor in this paper is enhanced by 430–1600 times. This work jumps out of the framework of directly modulating SAW in gyroscopes and provides an innovative scheme of the indirect modulations from the rotation-dependent PM on SAW, showing excellent performance and potential for angular velocity measurement in extreme environments.

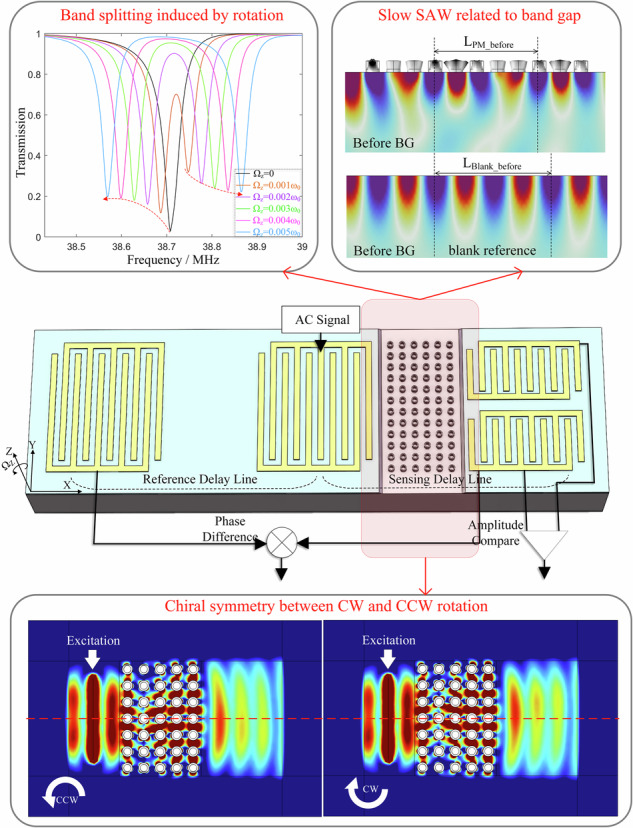

## Introduction

As one of the core devices of inertial navigation system, the gyroscope largely determines the performance and cost of inertial system, and is always the hot spot in the field of inertial navigation in various countries^1–3^. However, in some cases with extreme working environment (e.g., rocket launch, ammunition penetration, oil field drilling, etc.), the gyroscope is still required to work steadily with high accuracy under overloads of over 20,000 g or even 200,000 g^4,5^. Commonly, on-chip gyroscopes exist movable three-dimensional suspension structures, which would occur fracture, adhesion and particle contamination and eventually make the performance of gyroscopes deteriorate or even fail under overloads^6–8^. Therefore, it is difficult for on-chip gyroscopes to meet the demands of applications in such extreme working environments.

Under these circumstances, some all-solid-state surface acoustic wave (SAW) gyroscopes without suspension structure have been proposed. These SAW gyroscopes achieve the angular velocity measurement generally based on the rotation modulations on the properties of SAWs such as Rayleigh and Lamb waves in the solid substrates. They can be mainly categorized into two types according to their modulated properties, amplitude modulated type and phase velocity modulated type^9^. The amplitude-modulated SAW gyroscopes, in which the secondary SAWs were generated by the Coriolis effect and their amplitudes depended on the angular velocity, were first proposed by Kueasava et al.^10^. Thereafter, although several works on this kind of SAW gyroscopes have been reported^11–14^, their performances were not so satisfactory due to the weak secondary SAWs and inevitable lacks of temperature compensation structures^9,13,15^.

Instead of detecting the weak amplitudes of secondary SAWs, phase velocity-modulated SAW gyroscopes focused on the rotation effects on the phase velocity of SAW. In fact, as early as the end of the last century, scholars have realized that the Coriolis effect arisen from rotation could modulate the phase velocity of surface waves on elastic substrates^16,17^. With advances in sensing technology, the first prototype was reported until 2007 by Sang Woo Lee et al.^15^. They detected the SAW phase velocity on ST-cut quartz by SAW self-oscillators while obtaining a low sensitivity of 0.431 Hz/(deg/s). To find a substrate with more significant changes in SAW phase velocity, Sergey V. Biryukov et al. enumerated the SAW phase velocity versus rotation on typical piezoelectric substrates, concluding weak gyroscopic effect for pure piezoelectric substrates (shifts of only a few tens of meters per second under 0.1 relative rotation)^18^. Subsequently, the efforts were mainly devoted to the surface configuration of piezoelectric substrates to enhance velocity shifts. Wen et al. combined amplitude and phase velocity-modulated types with two SAW oscillators and achieved a high sensitivity of 119 Hz/(deg/s)^19,20^. They claimed that the secondary SAWs generated from one of the oscillators could interfere with the SAW in the other oscillator, thus changing the SAW velocity in the second oscillator. However, they have not explained how the interference occurs and affects the velocity, and how much it affects the velocity. Sang Woo Lee et al. suggested that rationally distributing metals on the substrate surface could enhance the Coriolis effect, thereby increasing the velocity shifts^21,22^. Unfortunately, even the best theoretical result of this method only shifts the phase velocity by about 100 m/s under 0.1 relative rotation. Since then, it seems that both amplitude and phase velocity-modulated types have come to a standstill. Although some new schemes like acousto-optical SAW gyroscopes emerged, they were in the theoretical stage^23^.

The weak gyroscopic effect in SAW gyroscopes is derived from the poor Coriolis effect of SAW itself under rotation, since the surface with metal dots merely features a particle velocity of 1.7e^−2^ m/s and a Coriolis force of 3.6e^−12^ N^13^. Actually, the SAW can be modulated and engineered more effectively in other ways. As one of the most promising ways, phononic metamaterial (PM) can control the SAW with high freedom by periodic arrays and local resonance units^24,25^, allowing confining or guiding SAW^26,27^. More importantly, the band structures flexibly designed by PM can also efficiently alter both the phase and group velocity of SAW^28–31^, inspiring a novel idea for phase velocity-modulated SAW gyroscopes. In our previous work^32^, we first reported a rotationally modulated PM based on the whispering-gallery modes (WGMs) of hollow-pillar resonators, which was formerly studied only for filters and waveguides^33–35^. The WGMs can be rotationally modulated because of their similarities to the working modes of hemispherical resonance gyroscopes, which feature the best overall performance among the Coriolis vibration gyroscopes^36–38^. Therefore, the WGMs could be the key to linking the velocity modulation by PM and the rotation. However, our previous work mainly focused on the precession phenomenon of WGMs themselves rather the band structures and velocity modulation.

In this paper, we link the phase velocity modulation by PM to the rotation, innovatively utilizing the hollow-pillar-based PM operated at WGMs as the vehicle for the Coriolis effect rather than SAW itself. The gyroscopic effects of the PM operated at WGMs are studied, as well as a novel SAW gyroscope based on the slow SAW in this PM is proposed. Both theoretical modeling and numerical simulation are employed to illuminate the gyroscopic effects and device performances. The following contents are arranged into four sections. In “Results”, the gyroscopic effects of the PM operated at WGMs are studied, including the band splitting, phase velocity shifting and rotation directionality. Besides, a novel SAW gyroscope is proposed, and its scheme, structure, principle, sensitivity and performances are respectively analyzed. In “Discussion”, we further discuss the improvement compared with current phase velocity-modulated SAW gyroscopes and the implementation plan. In “Conclusion”, we conclude this paper.

## Results

### Gyroscopic effects in phononic metamaterial

#### Band splitting under rotation

**Fig. 1:**
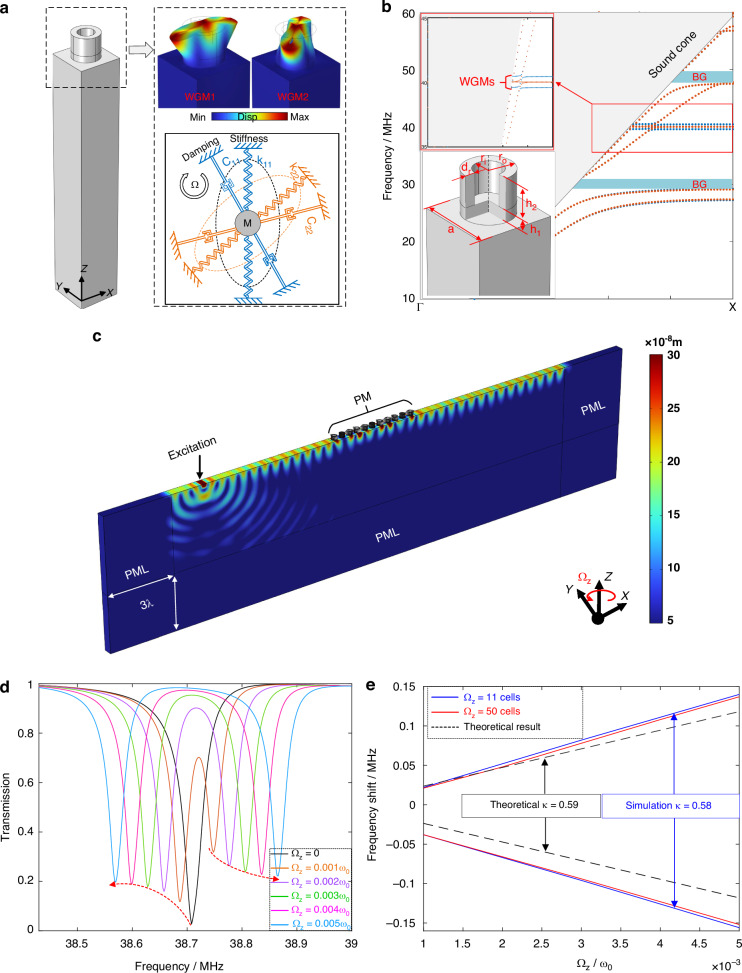
Theory, modeling, and results of WGMs band splitting induced by rotation. **a** Basic structure for a unit cell of PM array. The mode shapes and their coupling vibration system are shown in the dashed frame, respectively. **b** Band structure of the hollow-pillar-based PM under rotation and non-rotation. The structural parameters are summarized in Table 1. The gray zone represents the sound cone, and the blue and orange dotted lines refer to the bands under rotation or not, respectively. WGMs bands are enlarged in the red frame. **c** Simulation model for calculating transmission characteristics. The displacement field are captured near but out of WGMs. **d** Transmission characteristics of the PM with 11-cell length under increasing rotation, calculated from the model in (**c**). **e** Trends of the frequency shifts under growing rotation. The blue solid line, red solid line and the black dashed line denote the results of 11-cell PM, 50-cell PM and theoretical prediction, respectively

The PM consists of periodic units arranged in a square lattice, and each unit cell is composed by a hollow pillar and an infinite substrate, shown as Fig. 1a. This hollow-pillar unit is indispensable to our study due to its three key advantages. First, the hollow-pillar unit possesses full symmetry in the *x–y* plane, which ensures that its localized resonance modes appear in degenerate pairs with identical resonant frequencies and effective masses. Second, the hollow-pillar unit can introduce pairs of localized resonance modes with vibration orthogonality, named WGMs. In fact, the WGMs are very similar to the wine-glass modes which could be coupled to each other under the Coriolis effect (even sometimes considered as the same mode^39^), and only the second-order WGMs are considered in this paper because they are usually more accessible to excite than the high-order WGMs. Third, compared to the solid-pillar unit, the localized resonance modes of this hollow-pillar unit are more flexibly tunable across both low and high-frequency bandgaps without altering the overall band structure, facilitating structural design. Due to their degeneracy and vibration orthogonality, the free-vibration model of WGMs under rotation can be described by a typical 2-DOF vibration system, shown in Fig. 1a and expressed as^40^:

1 $$m\ddot{{\bf{q}}}+{\bf{C}}\dot{{\bf{q}}}+2{m}_{cori}{\boldsymbol{\Omega }}\dot{{\bf{q}}}-{m}_{cent}{\varOmega }_{z}^{2}{\bf{q}}+{\bf{K}}{\bf{q}}=0$$

where $${\bf{q}}={[\begin{array}{cc}{q}_{1} & {q}_{2}\end{array}]}^{T}$$ is the displacement vector along the principal axes of vibration, $${\boldsymbol{\Omega }}=[\begin{array}{cc}0 & -{\varOmega }_{z}\\ {\varOmega }_{z} & 0\end{array}]$$ is the matrix of rotation around z axis. $m$, ${m}_{cori}$ and ${m}_{cent}$ are the effective mass, Coriolis mass and centrifugal mass, respectively. $${\bf{C}}=[\begin{array}{cc}{c}_{11} & {c}_{12}\\ {c}_{21} & {c}_{22}\end{array}]$$ and $${\bf{K}}=[\begin{array}{cc}{k}_{11} & {k}_{12}\\ {k}_{21} & {k}_{22}\end{array}]$$ are the matrices of damping and stiffness. Considering the ideal case that the damping and stiffness are isotropic and aligned with the reference axis, ${c}_{11}={c}_{22}=c$, ${k}_{11}={k}_{22}=k$ while ${c}_{12},{c}_{21},{k}_{12},{k}_{21}=0$. The centrifugal force can be neglected because the rotation rate is much lower than the resonance frequency^41^. Thereby, the dynamic equations can be solved and the eigen frequencies of the vibration system are obtained as:

2 $$\omega =\sqrt{\frac{k}{m}}\pm \frac{{m}_{cori}}{m}{\varOmega }_{z}={\omega }_{0}\pm \kappa {\varOmega }_{z}$$

Equation (2) indicates that the WGMs split linearly with rotation and oppositely into two branches, where ${\omega }_{0}=\sqrt{k/m}$ is the initial eigen frequency without rotation and $\kappa ={m}_{cori}/m$ denotes the splitting factor. The ${m}_{cori}$ and $m$ can be obtained by the integrated normalized vibration parameters of WGMs over the volume of the hollow pillar^42^, such that ${m}_{cori}=\int \rho ({\phi }_{x1}{\phi }_{y2}-{\phi }_{y1}{\phi }_{x2})dV$ and $m=\int \rho ({\phi }_{x1}^{2}+{\phi }_{y1}^{2}+{\phi }_{x2}^{2}+{\phi }_{y2}^{2})dV/2$. Wherein ${\phi }_{x1}$, ${\phi }_{y1}$, ${\phi }_{x2}$ and ${\phi }_{y2}$ are the normalized displacements along *x* and *y* axis of WGM1 and WGM2 respectively (the displacement along *z* axis is too small and therefore neglected), and $\rho$ is the mass density of the hollow pillar. Therefore, the splitting factor can be further expressed as Eq. (3):

3 $$\kappa =\frac{2\int ({\phi }_{x1}{\phi }_{y2}-{\phi }_{y1}{\phi }_{x2})dV}{\int ({\phi }_{x1}^{2}+{\phi }_{y1}^{2}+{\phi }_{x2}^{2}+{\phi }_{y2}^{2})dV}$$

To verify the split of WGMs and further investigate the influences on PM, the finite element method (FEM) is employed to construct and calculate the PM models under rotation (in COMSOL Multiphysics 6.0). The material chosen for both the hollow pillars and substrate is single-crystal silicon, with mass density of 2329 kg/m^3^, Youngs modulus of 170 GPa and poison ratio of 0.28. And the structural parameters of the unit cells are optimized to approach mode match (minimizing the frequency difference of WGMs, 0.02 MHz in this paper), summarized in Table 1. Figure 1b shows the band structure along the direction of $\varGamma {\rm X}$ within the first Brillouin zone of the optimized unit cell. The gray zone represents the sound cone, where the SAW is leaky. The blue and orange dotted lines below the sound cone refer to the SAW bands under rotation or not, respectively. A pair of flat bands lie around 40 MHz are extra introduced by the hollow pillar, representing WGMs. Notably, the WGMs are designed in SAW bands rather than in bandgaps (BGs) like previous works^32–35^. This is because the flat dispersion indicates slow propagation velocity (both group and phase velocity) and high-energy localization, and these features determine that WGMs are more efficient and practical to store and absorb energy in SAW bands than transmit SAW in bandgaps. It can be seen that rotation rarely affects the dispersion curves and band structure except for WGMs (enlarged in the inset). The dispersion curves of WGMs split obviously into two branches under rotation, which is consistent with the prediction of the dynamic model above.

**Table 1:** Structural and characteristic parameters of PM unit cells

Parameters	Value
Material	Single-crystal silicon
Lattice constant (*a*)	50 µm
Height of hollow pillar (*h*_*1*_ + *h*_*2*_)	0.55*a*
Thickness of the bottom of hollow pillar (*h*_*1*_)	0.15*a*
Outside radius of hollow pillar (*r*_*o*_)	0.32*a*
Inside radius of hollow pillar (*r*_*i*_)	0.22*a*
Effective mass at WGMs ($m$)	2.64e^−8^ kg
Coriolis mass at WGMs (${m}_{Cori}$)	1.56e^−8^ kg
Splitting factor ($\kappa$)	0.59

To further quantify the effects of rotation on the PM operated at WGMs, the transmission characteristics around WGMs of the PM with finite unit cells are simulated. Figure 1c shows the 3-D finite element model to simulate the transmission characteristics and its displacement field near WGMs, exhibiting obvious SAW behavior. Perfect match layer (PML) with thickness of triple wavelength and periodic boundary condition are applied to avoid reflection and reduce computation, respectively. The meshing scheme is shown in Supplementary Fig. 1. The excitation is realized by the boundary condition of specifying displacement, exciting the SAW but additionally generating the bulk wave. Fortunately, due to the absorption of PML, the additional bulk wave will not influent the surface displacement. The rotation conditions are implemented under Rotating Frame node, where the amplitude, direction, and rotation axis location of applied rotation can be set. In this simulation model, the rotation amplitude is scanned parametrically from 0 to 0.005${\omega }_{0}$ at 0.001${\omega }_{0}$ intervals, and the rotation axis is set as *z* axis located at the center of model (the specific steps to apply rotation in COMSOL are shown in Supplementary Fig. 2). However, it is worth mentioning that the location of the rotation center has no effect on the results, since the Coriolis effect is irrelevant to it.

The transmission spectrum under increasing rotation in a case where a row of PM consists of 11 cells (the transmission of the PM with a length over 10 cells tends to stabilize, shown as Supplementary Fig. 3) is shown in Fig. 1d. The transmission is calculated by comparing the average displacements over the defined area before and after the PM. As expected, the transmission valley splits into two valleys, and the frequency shift of both sides grow linearly with rotation (We also employ the transfer matrix method to validate the FEM results, the detailed calculation process is shown in Supplementary Validation, showing consistent frequency splitting and transmission attenuation). However, the split valleys are asymmetric. The transmission of downshifted valley builds up with rotation increasing while the upshifted valley is contrary. Besides, the symmetry center of the downshifted and upshifted valley is not where the initial valley is. These unexpected phenomena can be explained by the deaf-band effect^43^ and the coupling between WGMs. In fact, the ideal case where no frequency difference between WGMs can hardly achieve, and the frequency difference can only be minimized as much as possible. However, there is only one transmission valley appearing when no rotation. This results from the deaf-band effect, and only the WGM1 which is symmetry about the propagation direction of SAW can be excited. When rotation applied, it can be known from Eq. (1) that WGM1 is coupled to WGM2 under Coriolis effect, and the coupling term $2{m}_{cori}\varOmega \dot{q}$ is proportional to rotation. Therefore, the energy of WGM1 is gradually transferred to WGM2 with rotation increasing, and the transmission valley of WGM1 becomes shallower while the transmission valley of WGM2 starts to appear and gets progressively deeper. Notably, the transmission valley of WGM2 upshifts from where the initial frequency of WGM2 rather than WGM1. And these are why the split valleys are asymmetric.

Figure 1e compares the trends of the frequency shifts under growing rotation in different cases. The blue and red solid lines denote the simulation results of 11-cell PM (the shortest effective length according to Supplementary Fig. 3) and 50-cell PM (the longest length considered in this paper), respectively, showing obviously linear relationship between the angular velocity and the frequency shift. These two nearly overlapping lines also demonstrate that the length of PM has barely effects on the frequency shift. The theoretical result for infinity unit cells is calculated by Eq. (3) and the normalized vibration parameters can be extracted from FEM (summarized in Table 1). Compared with the theoretical result (black dashed line), the splitting factor $\kappa$ of simulation result 0.58 slightly differs from the theoretical $\kappa$ of 0.59, and the symmetry center of the simulation result also shifts from the theoretical result. The deviations between the two results are because that the derivation of the theoretical result is under the ideal assumptions of the isotropic damping and stiffness while the simulation can only approach but not fully achieve these conditions. However, it still implies the high agreement between the two results, which verifies the correctness and rationality of both the theory and simulation from the side.

#### Phase velocity within PM

Due to the locally resonant BG additionally induced by WGMs, the group and phase velocity of SAW whose frequency is near WGMs are allowed to change. Compared with group velocity, the phase velocity is more commonly employed in SAW sensors, and is focused in this paper. The phase velocity of SAW can be extracted from frequency domain simulation by

4 $${v}_{PM}=\frac{{c}_{Rayleigh}Lengt{h}_{PM}}{Lengt{h}_{Blank}}$$

where ${v}_{PM}$ and ${c}_{Rayleigh}$ are the phase velocity of SAW within PM and without PM, respectively. $Lengt{h}_{PM}=n{\lambda }_{PM}$ ($n$ can be an integer or not) is the length of PM and ${\lambda }_{PM}$ is the wavelength of SAW within PM. While $Lengt{h}_{Blank}=n{\lambda }_{Blank}$ has the same *n* to $Lengt{h}_{PM}$, and is determined from a blank model without PM. This method to calculate phase velocity of SAW has proven to be effective by comparison with experimental results^31^.

**Fig. 2:**
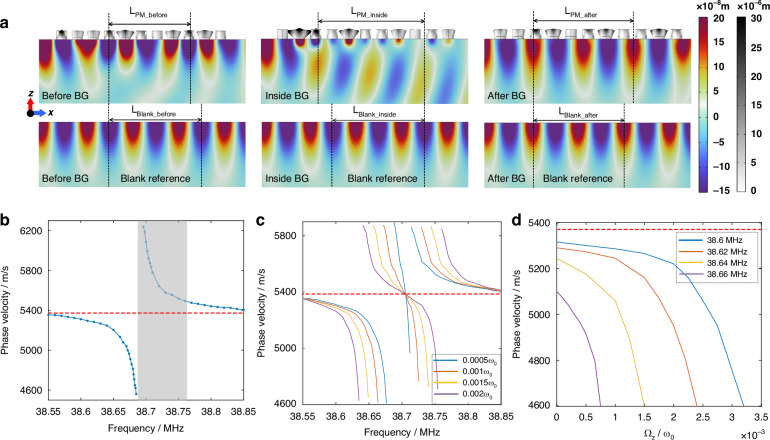
Varying phase velocity of SAW in PM. **a** Displacement fields of the PM-patterned substrate (11-cell PM), indicating the wavelength of SAW within PM. Three cases are captured, displacement fields when operated frequency is before, inside and after the BG. And corresponding displacement fields on a blank substrate are considered as references. **b** Phase velocity of SAW within PM near WGMs band, calculated by continuously extracting data from **a** and substituting them into Eq. (4). Gray region represents the BG of WGMs (region with less than half transmission). And the red dashed line indicates the phase velocity on blank substrate. **c** Phase velocity near WGMs band under increasing rotation. **d** Relationship between increasing rotation and the phase velocity of different operated frequencies

For specific demonstrations of the varying phase velocity in the area of PM and the method to extract it, Fig. 2a captures the displacement fields of both the PM-patterned and blank (as references) substrates before, inside and after the BG of WGMs, respectively (Supplementary Video 1 displays the simulation results of SAW propagating on the PM-patterned substrate for these three cases). The displacements of PM and substrate are displayed by two kinds of colormaps due to the relatively large differences. $Lengt{h}_{PM/Blank}$ for $n=2$ are labeled in corresponding cases. For the case before the BG, it can be seen that $Lengt{h}_{PM\_before}$ is shorted than $Lengt{h}_{Blank\_before}$ for the same frequency of SAW, indicating that the phase velocity of SAW within PM slows down. The slow SAW benefits from the strong excitation of WGMs, the displacement within PM is also consequently more concentrated on the substrate surface. For the case inside BG, however, the Rayleigh-like displacement field is mixed with the bulk displacement. Although longer $Lengt{h}_{PM\_inside}$ is obtained, it is attributed to a combination of modes above sound line. The fast phase velocity is likely to result from the average of those mixed modes^31^. Finally, for the case after BG, Rayleigh-like displacement is back to dominance, and $Lengt{h}_{PM\_after}$ gradually converges to the blank reference.

By continuously extracting $Lengt{h}_{PM/Blank}$ from the displacement fields like Fig. 2a and substituting them into Eq. (4), the phase velocity within PM near WGMs can be calculated and plotted as Fig. 2b. The gray region represents the BG (region with less than half transmission) induced by WGMs and the red dashed line marks the phase velocity of SAW on a blank substrate (${c}_{Rayleigh}=5355\,{\mathrm{m/s}}$). It is shown that the phase velocity near WGMs features strong dispersion. For the region before the BG, slower and slower SAW is presented with the frequency approach to the BG, and the slowest SAW reaches about 4600 m/s. However, for the region inside the BG, the fast SAW appears with a fastest speed of 6200 m/s, and the SAW in this region is very weak. Subsequently, the phase velocity gradually slows down with the frequency increasing, back to the reference velocity finally in the region after the BG. Figure 2a, b describes varying phase velocity in qualitative and numerical perspectives, respectively, showing great agreement and mutual support. Specifically, Fig. 2a provides the mechanism explanations for the varying phase velocity behavior, and Fig. 2b complements the trend of continuous changes in phase velocity.

As mentioned above, rotation can linearly induce the band splitting of WGMs. Therefore, it is predictable that the behavior of varying phase velocity related to WGMs could also be influenced by rotation. Figure 2c plots the phase velocity near WGMs under different angular velocities by the same method as Fig. 2b, and the phase velocity exhibits significant rotational dependence. Compared with the no-rotation case in Fig. 2b, an extra region of varying phase velocity is introduced between the original slow-SAW and fast-SAW regions. Different from the separated slow-SAW and fast-SAW regions, the rotation-introduced regions allow for continuous changes from fast to slow SAW. Analyzed in conjunction with Fig. 1d, it is recognized that the extra introduced regions appear between two split bands. Since the fast/slow SAW always appears inside/before the BG, the rapidly alternating BGs result in the continuous changes from fast to slow SAW. With the increasing angular velocity, the split WGMs and BGs linearly shift to opposite sides. Therefore, the separated slow/fast-SAW regions also linearly shift, with the continuously varying regions getting wider. The continuously varying curves of phase velocity under different angular velocities always intersect at a point which is the center of split bands.

Considering the modulation of the slow/fast-SAW regions by rotation, the phase velocity of SAW for a certain frequency will also be affected by rotation. Figure 2d plots the tends of the phase velocity at different operated frequencies (near but before BG, e.g., 38.6 MHz, 38.62 MHz, 38.64 MHz, and 38.66 MHz) with the angular velocity increasing. It is shown that the varying phase velocity transition from a slow-changing region to a fast-changing region under increasing rotation when the operated frequency is away from BG (like 38.6 MHz). This results from the gradually steeper dispersion with frequency and the linear shifts of BG with rotation. And as the operated frequency gradually approaches BG, the slow-changing region fades away (like 38.62 MHz and 38.64 MHz) until only the fast region left (like 38.66 MHz). The variable range of phase velocity is limited by the minimum phase velocity (4600 m/s for the determined PM) and could be further reduced by optimizing PM. Preferably, the phase velocity of SAW in PM can be modulated by about 450 m/s within a relative angular velocity of 0.7e^−3^. Therefore, the phase velocity within PM operated at WGMs is significantly sensitive to rotation, and it can be employed to develop a novel high-sensitivity SAW gyroscope.

#### PM under CCW and CW rotation

Although rotation can induce the band splitting of WGMs as demonstrated above, the band splitting is only dependent on the rotation rate regardless of the rotation direction. Specifically, the independence from rotation direction is reflected in the facts that both the band structures and the transmission characteristics under counterclockwise (CCW) and clockwise (CW) rotation are the same (Supplementary Fig. 4 shows the transmission curves under CCW and CW rotation). Accordingly, the varying phase velocity dependent on WGMs band will be unaffected by the rotation direction either.

**Fig. 3:**
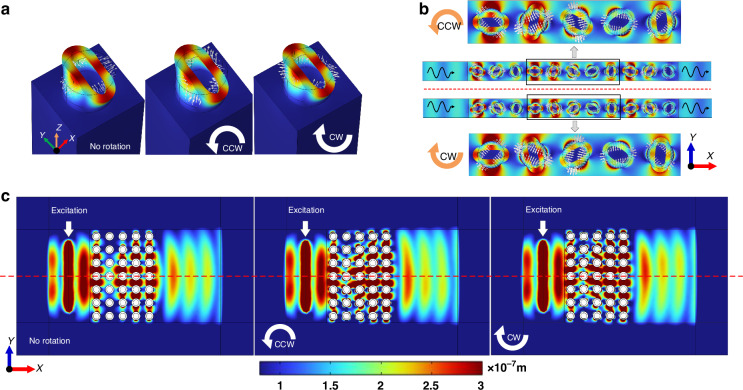
PM under CCW and CW rotation. **a** Mode shapes of WGM1 under no rotation, CCW rotation and CW rotation, respectively. The white arrows are the particle velocity vectors around the pillar, indicating vibration trends of WGM1. **b** Displacement fields of 11-cell PM under CCW and CW rotation. Several center units in the black frame are enlarged for clearer presentation. **c** Displacement fields of a complete 3-D model under no rotation, CCW and CW rotation. The red dashed line is the midline of the model structure, showing opposite asymmetry between CCW and CW cases

Actually, despite the rotation direction has no effects on the collective properties of WGMs (e.g., the band structure and transmission), it can exactly influence the mode behavior of each unit cell. Figure 3a displays the mode shapes and vibration trends of WGM1 in three cases of no rotation, CCW rotation and CW rotation, respectively, where the white arrows are the particle velocity vectors around the pillar. It can be seen that WGM1 in the three cases vibrate in different trends even they have the same mode shape. When under no rotation, the WGM1 keeps reciprocating vibration straightly along x and y axis. Differently, when the rotation is applied, the directions of velocity vectors occur deflections under the effects of Coriolis forces, thus the vibration of WGM1 involves an additional rotation motion. And the inverse Coriolis forces under CCW and CW rotation result in the opposite rotation trends (CCW/CW rotation induces CW/CCW-rotation trend of WGM1). Therefor, the mode behaviors of WGM1 under CCW rotation and CW rotation are chiral symmetric, so as WGM2. It is worth noting that this kind of additional rotation motion is different from the precession phenomenon demonstrated in our previous work^32^. The additional rotation motion is a kind of mode behavior rapidly occurring within each vibration period, while the precession phenomenon is essentially a slow accumulation of inertial delays linearly related to the angular velocity integral.

When we extend the case from the modal analysis of a single cell to the frequency domain analysis of a finite-cell PM array, the same results are obtained. Figure 3b shows the displacement fields of a PM with 11-cell length under SAW excitation and CCW/CW rotation, the model and boundary conditions (except the rotation direction) are the same as the descriptions to Fig. 1c. For clearer presentation, the displacement fields and particle velocity vectors of several center units in the black frame are enlarged. It is obvious that under a rotation with given direction (whether CCW or CW), each cell of PM has the same rotation trend even they vibrate with different phases. In comparing the two cases, apart from the overall opposite rotation trends, each cell vibrates with the same phase to its corresponding cell, also exhibiting the chiral symmetry between the two cases. These results are not only consistent with the modal analysis of a single cell, but also established for the PM with arbitrary length.

Although the chiral symmetry results in the coincidence of WGMs bands or transmission curves between CCW and CW cases, it implies the asymmetry in vibrating PM. Figure 3c shows the displacement fields of the complete 3-D model rather the periodically equivalent models (5-cell PM to reduce the computation), so that the distributions of transmissive SAW can be visually exhibited (Supplementary Video 2 displays SAW propagates in the 3-D model under no rotation, CCW rotation and CW rotation, respectively). The red dash line refers to the structure midline and divides the model into upper and lower parts. When no rotation applied, the PM vibrating reciprocally along the x and y axes is symmetrical about the midline, the transmissive SAW is also symmetrical about the midline as expectation. However, when under rotation, the unidirectional rotation trend introduced in PM vibration breaks the symmetry. For the CCW case, the CW-rotation trend of each cell provides extra momentum along the -y axis to the transmissive SAW. As a result, the symmetry of the transmissive SAW is also broken, and the transmissive SAW is more distributed in the lower part of the model. On the contrary, the transmissive SAW is more distributed in the upper part for the CW case. Therefore, the rotation directions can be distinguished by comparing the amplitudes between the upper and lower parts.

### Device design and performance

#### Device design and principle

**Fig. 4:**
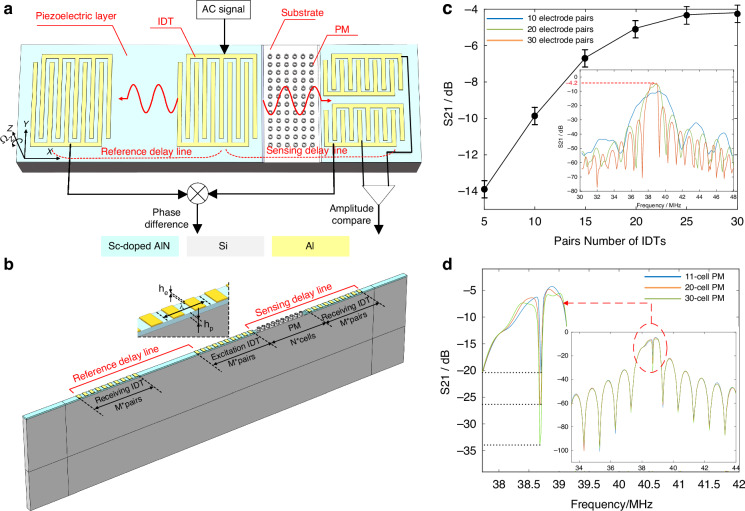
Design proposal of a new SAW gyroscope based on PM and its characteristics. **a** Proposed phase velocity-modulated SAW gyroscope, consisting of two sensing delay lines (containing PM) and a reference (blank) delay line. Based on the gyroscopic effects of PM, angular velocity can induce a rate-dependent phase difference between reference and sensing delay lines, as well as a direction-dependent amplitude difference between upper and lower sensing delay lines. **b** Simulation model of proposed gyroscope. The BIDTs on the 40% Sc-doped AlN piezoelectric film are employed. The excitation/receiving electrodes of IDT are set with voltage/floating and ground terminals. **c** ILs of reference delay line with different numbers of electrode pairs. **d** ILs of sensing delay line with different length of PM

Based on the gyroscopic effects on the hollow-pillar PM demonstrated above, a novel SAW gyroscope is proposed and designed as Fig. 4a. The SAW gyroscope consists mainly of two kinds of delay lines, one of which containing PM serves for sensing while the other for reference. A bidirectional interdigital transducer (BIDT) connected to a continuous AC signal at fixed detection frequency is utilized to generate SAWs propagating along the positive and negative *x* axis. The SAW propagating along the positive *x* axis within the sensing delay lines interacts with PM to excite WGMs. And the phase velocity and amplitude of SAW within the area of PM are modulated by the rotation rate and direction around *z* axis, respectively. While the SAW propagating along the negative *x* axis keeps independent from the rotation and can be employed as the reference and temperature compensation signals. Therefore, there appears a rotation rate-dependent difference between the delay times of reference and sensing delay lines, as well as a rotation direction-dependent difference between the amplitudes of upper and lower sensing delay lines. Specifically, the differences of delay times and amplitudes can be accurately measured by lock-in and differential amplifiers, thereby the angular velocity is detected.

The delay lines are the core components of the device for sensing occurrence, references generation, and interconversion between electrical signals and SAWs. The BIDTs are used in the proposed gyroscope so that the same initial SAWs can be simultaneously generated in both the reference and sensing delay lines. Rather than on typical piezoelectric substrates (e.g., LiNbO_3_ and quartz), the BIDTs in this gyroscope are constructed on a silicon substrate deposited with a layer of piezoelectric film. This is because that typical piezoelectric substrates always feature anisotropy, which makes it difficult to fabricate highly symmetrical geometries such as hollow-pillar-based PM in this paper. Besides, material anisotropy can deteriorate the degeneracy and weaken the Coriolis-induced coupling between WGMs. As a result, the rotation effects on WGMs will get weaker or even disappear. In comparison, silicon substrate possesses relatively good isotropy and is widely applied for micro gyroscopes based on degenerated modes (e.g., hemispherical resonance gyroscopes and ring resonance gyroscopes). However, silicon is non-piezoelectric material and a layer of piezoelectric film is necessary for a SAW device. Considering the anomalously outstanding piezoelectricity and piezoelectric coefficient, the Sc-doped AlN (ScAlN, 40% Sc concentration) film is chosen as piezoelectric film, and the material constants refer to a typical report^44^. For the BIDTs with periodic interdigital electrodes, every period is evenly distributed with two electrodes, one of which is connected to an AC power source or output circuit (excitation or receiving IDT) while the other is grounded. Commonly, the length of every period equals to the wavelength of Rayleigh waves and determines the operating frequency of the BIDTs at Rayleigh mode. Therefore, the center frequency of BIDTs can match the WGMs of PM by adjusting the length of every period.

Different from the model to calculate the transmission characteristics of PM in Fig. 1c, the model of delay line is required to taking the electrical characteristics into account. In detail, the SAW is excited by the inverse piezoelectric effect and detected by piezoelectric effect in the area of IDTs. Due to the significant difference in length between the apertures of IDT electrodes (commonly $\ge 50{\lambda }_{SAW}$) and the lattice constant of PM, the model for the reference and sensing delay lines can also be simplified and equivalent to a row by applying the periodic boundary condition, shown as Fig. 4b The reference and sensing delay lines are respectively located on the left and right sides of the model, and the blank area for reference delay has the same length as the PM area for sensing. The silicon substrate, silicon PM and aluminum electrodes are treated as the linear elastic materials based on the small-deformation hypothesis, while the Sc-doped AlN film is treated as the piezoelectric material. The sides and bottom of the model are provided with PMLs to absorb reflections from the boundaries. The electrodes of excitation IDT are set with voltage and ground terminals, while the electrodes of receiving IDT are set with floating and ground terminals.

Based the model built above, the key characteristics of the reference and sensing delay lines, the insertion losses (ILs), can be extracted respectively by the expression $20\,\log ({V}_{exc}/{V}_{flo})$, where ${V}_{exc}$ and ${V}_{flo}$ are the voltages of excitation and floating terminals. We consider the case where excitation and receiving IDTs own the same number of electrode pairs. When varying the number of electrode pairs and PM cells, the ILs of reference and sensing delay lines are respectively shown in Fig. 4c, d. For the reference delay line, Fig. 4c displays the tendency of maximum IL versus increasing number of electrode pairs, and the IL around the center frequency is plotted in the insert. It indicates that increasing number of electrode pairs results in slower improvement in the maximum IL and narrower bandwidth. The boost of IL is highly limited after the number of electrodes exceeds 30 pairs. Therefore, excitation and receiving IDTs are set as 30 pairs with a maximum IL of −4.2 dB around 38.5 MHz, and this configuration is also valid in the receiving IDT of sensing delay line. For the sensing delay line, the introduction of PM and the frequency match design bring about a sharp decrease of IL around the center frequency of delay line, shown as Fig. 4d. Compared with the IL of reference delay line, the PM barely affects the IL over a large frequency range (32–47 MHz) except where PM works at WGMs. This benefits from the fact that the WGMs is designed in SAW bands rather than bandgaps of PM (Fig. 1b). Therefore, even if the length of PM is increased, only the IL falling in the BG of WGMs consistently decreases, while the IL at the other frequency rarely changes. Notably, although the IL near the BG changes far less drastically than the IL within the BG, it is more sensitive to the length of PM than those far from the BG. This because the area near the BG of WGMs corresponds to where the phase velocity changes, and the loss of SAW owning varying phase velocity is more susceptible to the length of PM. The key configurations of the models for reference and sensing delay lines are concluded in Table 2.

**Table 2:** Key configurations of the model for reference and sensing delay lines

Configurations	Value
Center wavelength of SAW (λ)	124 µm
Width of electrode	λ/4
Thickness of electrode	100 nm
Thickness of the piezoelectric layer	λ/10
Number of electrode pairs	30
Number of PM cells	$\ge$11

#### Sensitivity and device performance

For a sensor based on SAW delay line, the phase velocity of SAW is evaluated by accurately measuring the time delay or the phase of output response^45^. In fact, the measurement based on phase yields accuracies of 150–1500 times higher than based on time delay^46^. The phase of the output response can be expressed as $\varphi =2\pi {f}_{\!0}\tau$, where ${f}_{\!0}$ refers to the frequency of propagating SAW and $\tau$ denotes the delay time. In the case of the reference delay line, the delay time can be expressed as ${\tau }_{r}=L/{v}_{SAW}$, where $L$ is length of the delay line and ${v}_{SAW}$ is the phase velocity of propagating SAW. While in the case of the sensing delay line, the delay time is written as ${\tau }_{s}={L}_{no\_PM}/{v}_{SAW}+{L}_{PM}/{v}_{PM\_SAW}$, where ${L}_{no\_PM}$ and ${L}_{PM}$ are the lengths of the area without and with PM respectively (${L}_{no\_PM}+{L}_{PM}=L$), and ${v}_{PM\_SAW}$ refers the phase velocity of SAW within PM. Therefore, the phase difference between two delay lines can be expressed as:

5 $$\varDelta \varphi =2\pi {f}_{\!0}({\tau }_{s}-{\tau }_{r})=\frac{-2\pi {f}_{\!0}{L}_{PM}\varDelta v}{{v}_{SAW}({v}_{SAW}+\varDelta v)}$$

It is indicated that the phase difference only occurs and accumulates in the area of PM. Therefore, the length of PM is the effective length of sensing delay line. And as the phase velocity difference between ${v}_{PM\_SAW}$ and ${v}_{SAW}$, $\varDelta v$ is subjected to the angular velocity and usually far smaller than ${v}_{SAW}$. Hence, the sensitivity of the proposed SAW gyro can be expressed as:

6 $$S=\frac{\partial \varphi }{\partial {\varOmega }_{z}}\approx \frac{-2\pi \gamma }{{v}_{SAW}}\cdot \frac{\partial {v}_{PM\_SAW}}{\partial {\varOmega }_{z}}$$

where $\gamma ={L}_{PM}/\lambda$ is the relative length of PM compared with the SAW wavelength of the reference delay line. It is evident above that ${v}_{PM\_SAW}$ is a frequency-depended function under no rotation, and rotation causes the entire function to translate by a splitting factor of $\kappa ={m}_{cori}/m$ in the frequency domain. Therefore, the ${v}_{PM\_SAW}$ under rotation can be denoted as $V(f+\kappa {\varOmega }_{z})$, and the sensitivity for a certain operating frequency can be obtain as follows:

7 $${S}_{{f}_{0}}=\frac{-2\pi \gamma \kappa V^{\prime} ({f}_{\!0}+\kappa {\varOmega }_{z})}{{v}_{SAW}}$$

Equation (7) implies three factors contributing to the sensitivity, respectively splitting factor $\kappa$, relative length $\gamma$ and dispersion coefficient of SAW velocity $V^{\prime} ({f}_{\!0}+\kappa {\varOmega }_{z})$. First, the splitting factor is determined by the design of PM and can be improved by degeneracy optimization of WGMs (reducing the frequency difference and enhancing the quality factor of WGMs). However, it is often unprofitable to expend too much effort on improving the splitting factor. The ideal splitting factor for the second-order WGMs is relatively constant, and too high-quality factor delivers only a limited improvement but will restrain the coupling of the SAW to the WGMs. Second, the relative length of PM is essentially the range of spatial integral for phase accumulation. Although extending the relative length can reduce the IL of sensing delay line to some extent, it will simultaneously linearly improve the sensitivity. Notably, this tradeoff is worthwhile, since every expansion of the relative length of 5 (the length of 10 cells) only sacrifices about 1-dB IL. Third, the dispersion coefficient of SAW velocity depends on the selection of the operating frequency, and the sensitivity is tunable by adjusting the operating frequency. Particularly, when the operating frequency approaches or keeps away from WGMs (fast/slow-changing region), the dispersion coefficient and sensitivity approximate the constants under small rotation. Furthermore, the closer operating frequency to the WGMs corresponds to the higher dispersion coefficient and sensitivity. Therefore, the operating frequency approaching WGMs is preferred.

**Fig. 5:**
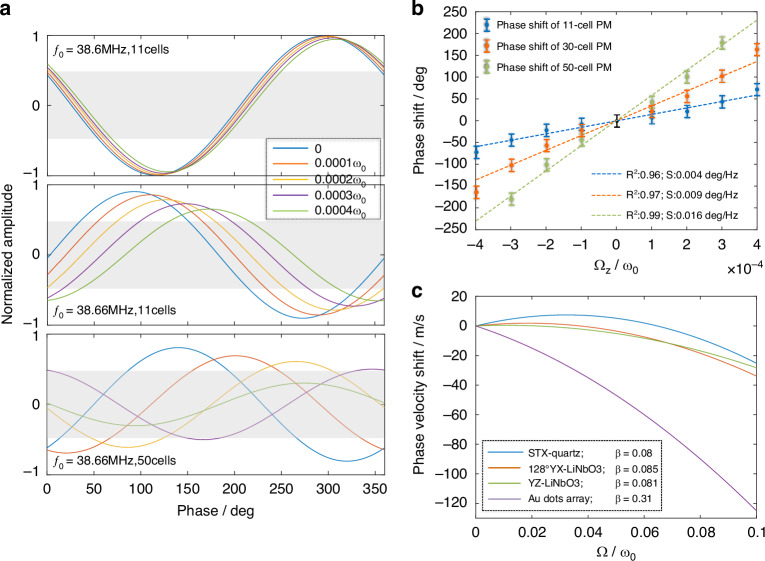
Performance of the proposed SAW gyroscope. **a** Amplitudes and phases of the outputs of delay lines under increasing angular velocity. The voltage amplitudes are normalized by the results of the reference delay line, and the phases are referenced to the excitation signal. Three representative cases are exhibited. **b** Curves of angular velocity vs. phase shifts under different length of PM. **c** Gyroscopic gain factors of existing phase velocity-modulation gyroscopes, enhanced by 430–1600 times in proposed gyroscope

Figure 5a exhibits the amplitudes and phases of the outputs of delay lines under increasing angular velocity for three representative cases, operated at 38.6 MHz with 11-cell PM, operated at 38.66 MHz with 11-cell PM and operated at 38.66 MHz with 50-cell PM. The voltage amplitudes of floating terminals are normalized by the results of reference delay line, and the phases are referenced to the signal of excitation terminal (the zero-phase point on the horizontal axis corresponds to the initial phase of excitation signal). The area where amplitudes are below half are marked by gray, and the curves entirely falling into the gray area are the outputs inside BG. The top diagram of Fig. 5a is plotted for the case that the operated frequency is away from WGMs (in slow-changing region) with relatively short PM. In this case, due to the relatively low dispersion coefficient, the increasing rotation only results in small phase shifts and the amplitudes remain virtually unchanged. While in the case that the operated frequency is near WGMs (in a fast-changing region) with relatively short PM plotted as the middle diagram, the phase shifts with increasing rotation are markedly improved, which emphasizes the important role of the relatively high dispersion coefficient and the selection of operated frequency. Besides, the amplitudes tend to decrease under increasing rotation, implying that the splitting BG induced by rotation is gradually approaching the operated frequency. When keeping the operated frequency near WGMs but extending the length of PM (shown as the bottom diagram), the phase shifts are further increased as predicted owning to the accumulation of phase differences over longer distances. These results are in good agreement with Eq. (7). Unexpectedly, the amplitudes decay with the rotation increasing more rapidly even drop into BG. This may be due to the fact that the extended PM widens the bandwidth of BG. And the curve falling into BG shows an abrupt and irregular phase change which is an invalid output, resulting in a shorter sensing range. The phase shifts of the proposed SAW gyroscope operated near WGMs are plotted as Fig. 5b. The results all demonstrate excellent linearity, and the sensitivity increases with PM length as expected. When the sensing delay line has 50-cell PM, the gyroscope features a sensitivity of 0.016 deg/Hz with a range of $\pm$3e-4${\omega }_{0}$. The sensitivity can be further improved by extending the length and optimizing PM.

## Discussion

Different from the phase detection in our proposal, existing phase velocity-modulated SAW gyroscopes mostly measure the angular velocity by detecting the center frequency shifts of the SAW resonators. To compare the proposed and the existing phase velocity-modulated SAW gyroscopes, the gyroscopic gain factor $\beta =\varDelta v\cdot \omega /\varDelta \varOmega \cdot {v}_{SAW}$ (commonly to evaluate gyroscopic effects in phase velocity-modulated SAW gyroscopes^15,18,21,22^) is utilized. The gyroscopic gain factors of the common substrates of the existing SAW gyroscopes are summarized in Fig. 5c^18,47^. The gyroscopic gain factors are not significantly different among these piezoelectric substrates with free surface (*β* is about 0.08), and are relatively markedly improved for the piezoelectric substrates with Au dots array on surface (*β* is about 0.31). In comparison, the gyroscopic gain factor reaches about 133.4 (phase velocity shifts 500 m/s under 0.0007${\varOmega /\omega }_{0}$ rotation), it is enhanced about 1600 times over free-surface piezoelectric substrates and about 430 times over Au-dotted piezoelectric substrates, showing significant enhancements. Moreover, the slowest phase velocity obtained in this paper is 4600 m/s while the flat dispersion curves of WGMs theoretically feature slower or even near zero-phase velocity, showing considerable room to further reduce the velocity of SAW. This indicates a potential for working with weak frequency dependence while maintaining high sensitivity.

In the future practical experiments, there are three main issues to consider, including device fabrication, system building and temperature compensation. For the device fabrication, the Sc-doped AlN film can be deposited by the standard processes for magnetron sputtering, and the BIDTs can also be patterned by the standard processes for electron beam evaporation and lift-off. The potential challenge is the fabrication of hollow-pillar-based PM with an aspect ratio of 5.5. Fortunately, the deep reactive ion etching could be a good solution for the PM since it has been successfully applied to process similar pillar arrays with a high aspect ratio of up to 22^48^. For system building, the primary issue is the supply of excitation signals for the delay lines, considering the frequency dependence of the sensitivity. One feasible method is to employ a direct digital synthesizer to provide clean, continuous excitation signals with high resolution, such as Analog Devices AD9954 (with sub-hertz resolution)^49^. Another possible approach is to use a relatively broadband excitation signal and then extract the signals at the target frequency by means of signal processing. In addition, the detection of phase shifts and rotation directions is also need to consider in system building. The phase shifts can be demodulated in a lock-in amplifier with the output of reference delay line as the reference signal. And the rotation directions can be judged by comparing the amplitudes from the two sensing delay lines in a differential amplifier. As for temperature compensation, the temperature has two main impacts on the proposed SAW gyroscope, including the propagation velocity of SAW and the resonant frequency of WGMs. Regarding the impact of temperature on the SAW velocity, it can be compensated during the processes of phase demodulation and differential amplification owning to the differential design in device structure. As to the temperature effect on the WGMs resonant frequency, it is required to employed additional methods to compensation, such as digital compensation techniques (using digital filtering, mathematical compensation models, etc., to estimate and correct temperature drift based on pre-established algorithms), software compensation approaches (employing artificial intelligence methods such as neural networks, genetic algorithms, or numerical analysis for compensation), and test calibration methods, among others.

## Conclusion

In this paper, the gyroscopic effects of the hollow-pillar-based PM operated at WGMs are investigated for the first time, and a novel phase velocity-modulated SAW gyroscope is proposed according to these gyroscopic effects. The band of WGMs can linear split under increasing rotation with a splitting factor of 0.58, and the slow phase velocity depending on the location of WGMs band is consequently modulated by the rotation. Besides, the PM operated at WGMs features opposite rotation tends and chiral symmetry under CCW and CW rotation, resulting in asymmetric SAW distribution. Based on these gyroscopic effects of PM, we propose and design a novel SAW gyroscope, obtaining a sensitivity of 0.016 deg/Hz with a range of $\pm$3e-4${\omega }_{0}$. Compared with existing phase velocity-modulated SAW gyroscopes, the gyroscopic gain factor is enhanced by 430–1600 times. The performance of the proposed gyroscope can be further improved by extending the length for longer delay and optimizing PM to lower phase velocity, showing excellent potential for angular velocity measurements in extreme environments. Furthermore, utilizing PM instead of SAW itself to carry the Coriolis effect and sense angular velocity in this paper provides a new idea for SAW gyroscopes.

## Supplementary information

supplementary figs

supplementary validation for FEM

supplementray_video1_varying_phase_velocity

supplementray_video2_rotation_direction

supplementary_data1_Bandstructure

supplementary_data2_Transmission

supplementary_data3_Phase_Velocity

supplementary_data4_IL_reference

supplementary_data5_IL_PM

supplementary_data6_phase_shift

supplementary summary

## Data Availability

The simulation models and methods used to produce Figs. 1–5 are described throughout this paper, and the meshing scheme is shown in Supplementary Fig. 1. The band structure data and transmission data to produce Fig. 1 are provided in Supplementary Data 1 and Supplementary Data 2. The frequency splitting and transmission attenuation reflected by Fig. 1d are validated in Supplementary validation. The captured pictures in Fig. 2a are from Supplementary Video 1, and the phase velocity data under different rotation rates to produce Fig. 2b–d is provided in Supplementary Data 3. The simulation results of the complete 3-D model under different rotation directions as demonstrated in Fig. 3c are shown in Supplementary Video 2. The IL data obtained from the model in Fig. 4a, b and to produce Fig. 4c, d is provided in Supplementary Data 4 and Supplementary Data 5, respectively. The phase data to plot Fig. 5a, b is provided as Supplementary Data 6 accompanying this paper.
