# Supplementary material for: A novel gyroscope based on the slow surface acoustic wave in a phononic metamaterial
Source: Microsyst Nanoeng. 2024 Nov 14;10:169. doi: 10.1038/s41378-024-00787-1 (PMC11564662; doi:10.1038/s41378-024-00787-1)
Supplement: Supplementary file 1 — supplementary figs [file 41378_2024_787_MOESM1_ESM.docx]

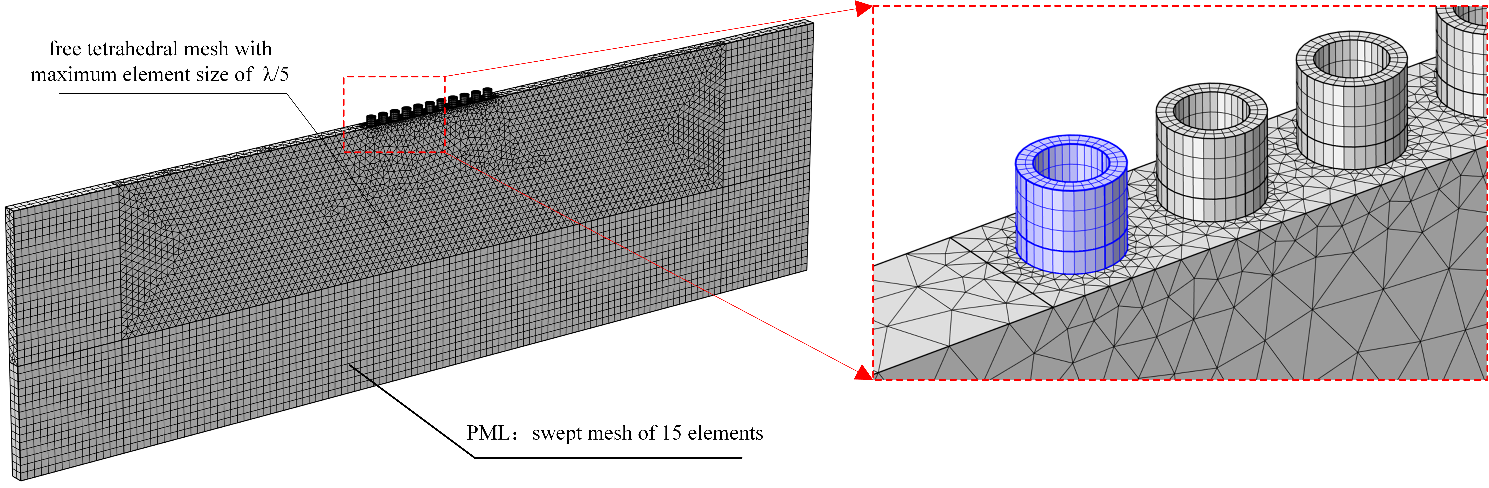


**Supplementary Fig.1 Meshing scheme example.** To keep hollow pillars consistency, a single hollow pillar is meshed first and copied to the others. For a symmetry pillar, first divide the top circular edges equally into multiple segments, subsequently mesh the top surface symmetrically based on these segments using free quadrilateral mesh, and finally sweep down. The main body of this model is meshed by free tetrahedral mesh, and the maximum element size is set as one-fifth of the minimum SAW wavelength. The PMLs are meshed by swept mesh with a distribution of 15 elements. The rest of the simulation models in this paper have similar meshing schemes.


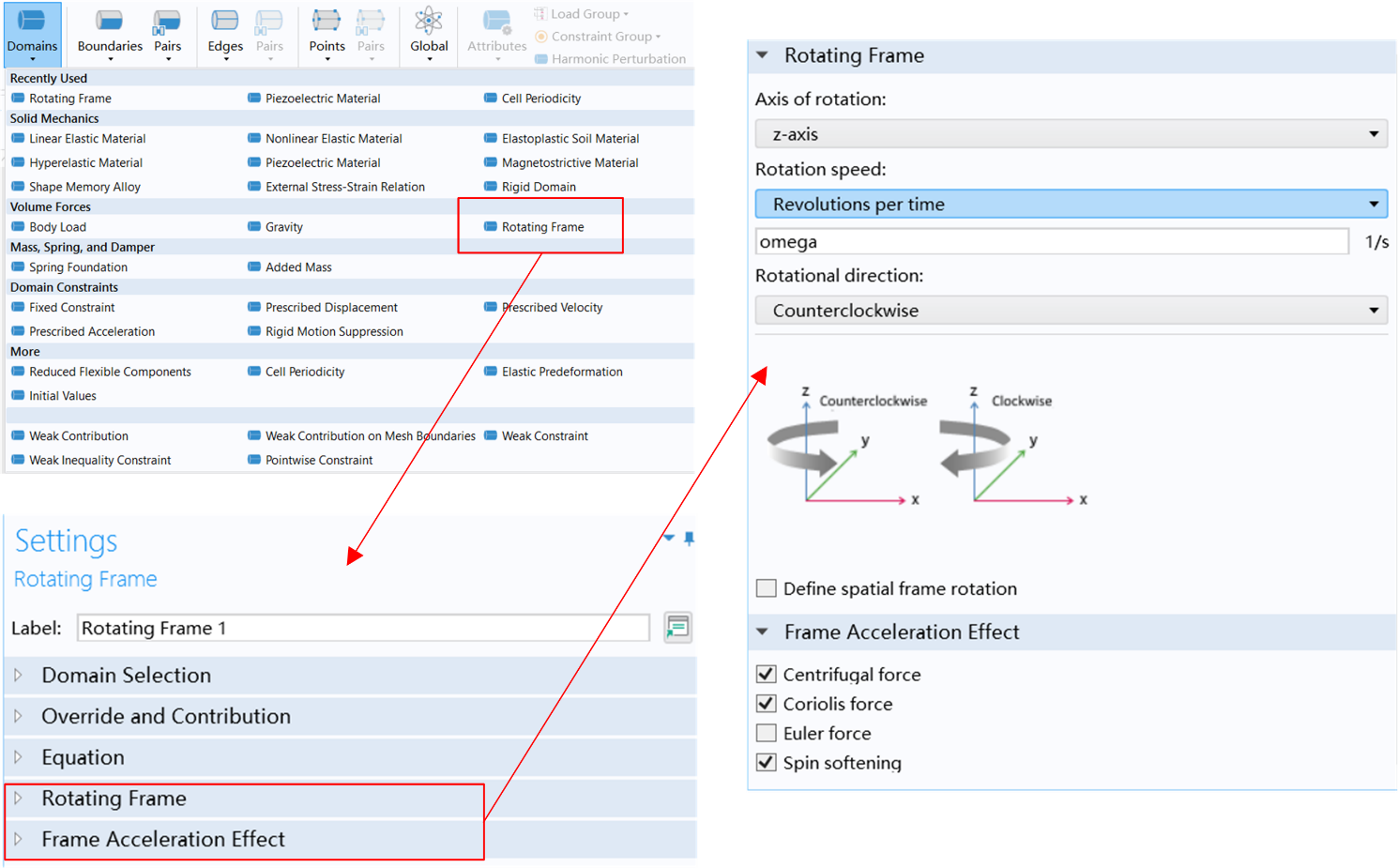


**Supplementary Fig.2 Method to apply rotation in COMSOL simulation.** Firstly, add Rotating Frame Domains under Structural Mechanics Module node to place the model in a rotating environment. Secondly, customize the boundary conditions for rotation in the settings of the Rotating Frame Domains. In this step, the key settings include rotating frame and frame acceleration effect. In the sub-node of rotating frame, the z axis locating at the center of model is set as the rotation axis, and the “omega” of rotation speed is a defined variable. In the sub-node of frame acceleration effect, check the rotation effects to be considered including centrifugal force, Coriolis force and spin softening (Euler force is not considered because it exists only for time-varying rotational speeds). Finally, scan “omega” parametrically from 0 to 0.005$\omega_{0}$ at 0.001$\omega_{0}$ intervals under Study node to calculate results for different rotation speeds.


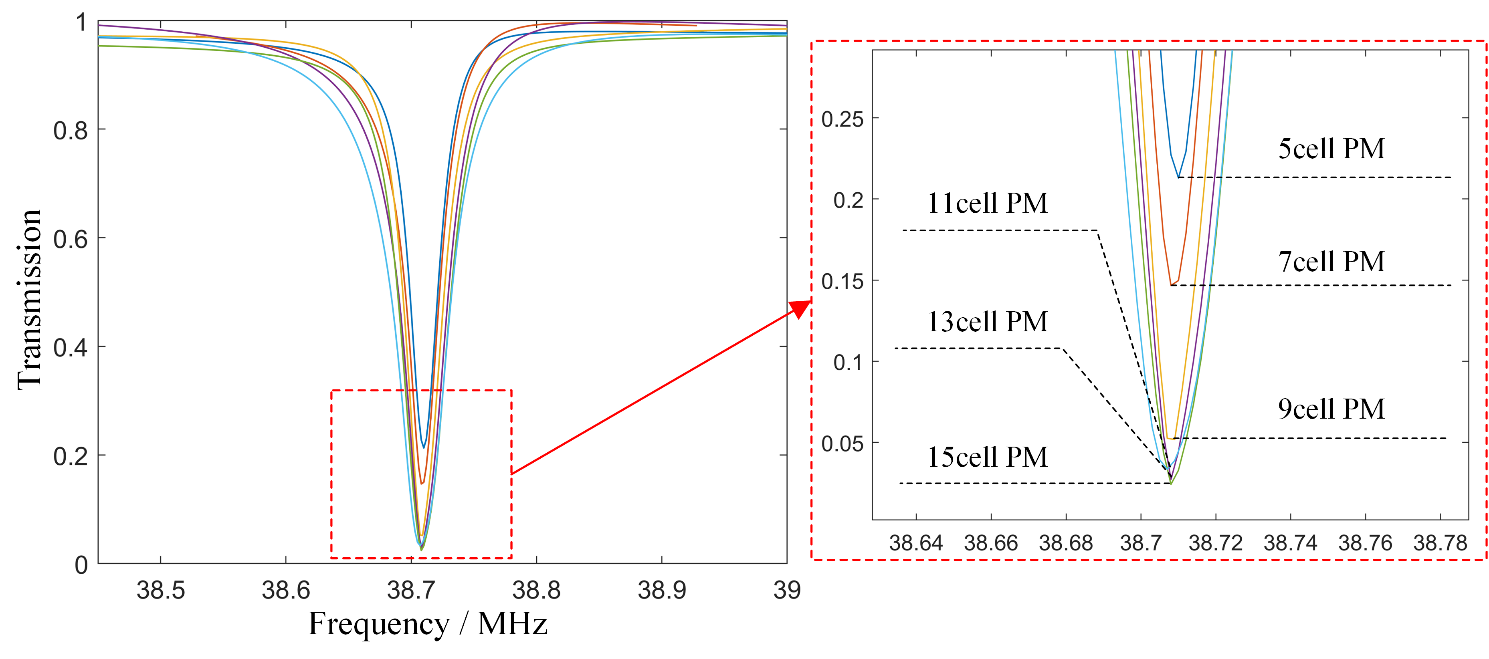


**Supplementary Fig.3 Transmission characteristics of PM with different lengths.** When the PM with a length of 5 cells, its transmission curve shows relatively high transmission rate and narrow bandwidth. With the length of PM increasing, the transmission valley is getting deeper and wider, indicating lower transmission rate and broader bandwidth. And these influences from increasing length of PM are gradually stabilized when the length over 11 cells.


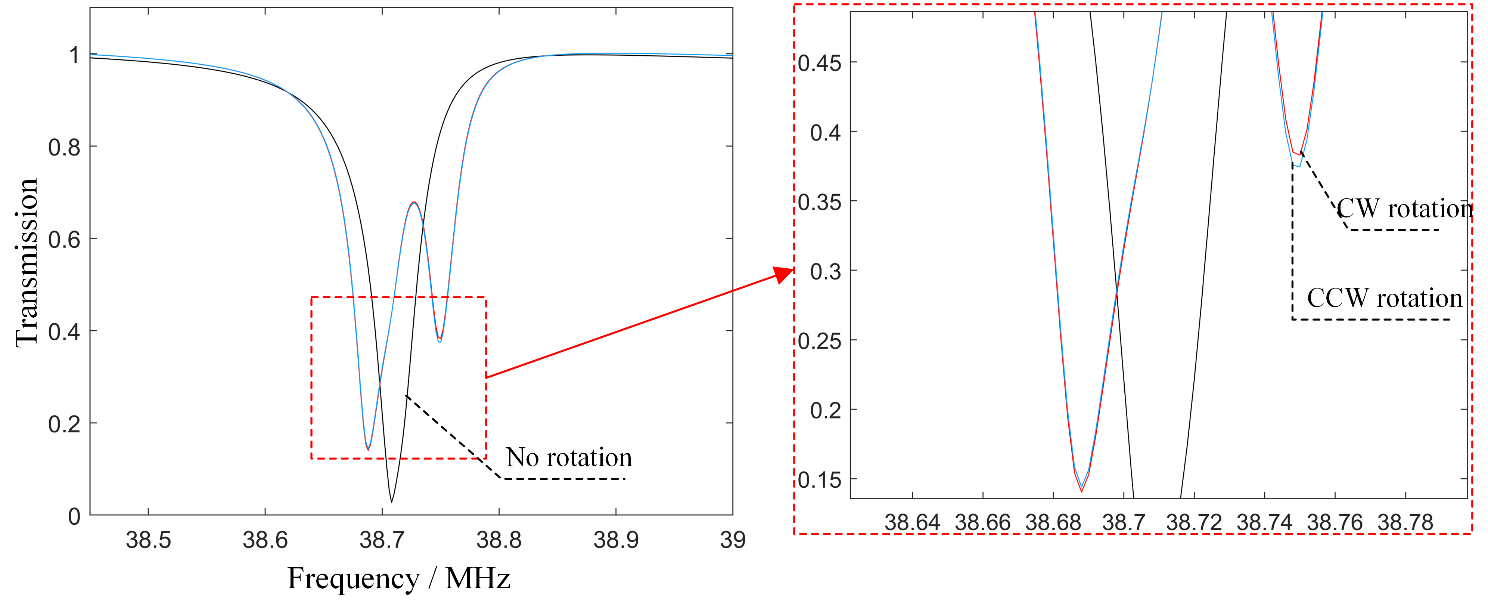


**Supplementary Fig.4** **Transmission characteristics of PM under CCW and CW rotation.** When 11-cell PM under CCW and CW rotation with a rate of 0.001ω, their transmission curves almost overlap. Even though there are some very tiny differences, they are only due to unavoidable mesh errors. Therefore, rotation direction has no effect on band splitting and transmission characteristics of WGMs.
