# Supplementary material for: A novel gyroscope based on the slow surface acoustic wave in a phononic metamaterial
Source: Microsyst Nanoeng. 2024 Nov 14;10:169. doi: 10.1038/s41378-024-00787-1 (PMC11564662; doi:10.1038/s41378-024-00787-1)
Supplement: Supplementary file 2 — supplementary validation for FEM [file 41378_2024_787_MOESM2_ESM.docx]

**Equivalent model of PM under rotation**.

In modeling PMs featuring locally resonant modes by the theoretical approaches such as the PWEM and the TMM, it is commonplace to employ an equivalent representation of periodically distributed spring-mass systems. Here, we equivalently model the two-dimensional array of hollow pillars on a thick substrate, as presented in the manuscript, as a one-dimensional arrangement of spring-mass systems along a slender string in the x-z plane, shown as Fig.1. To incorporate rotational effects, each mass element is considered as a two-degree-of-freedom second-order system in the x-y plane. It is noteworthy that while such an equivalent model simplifies specific aspects of the original model, such as the two-dimensional distribution and the thick substrate, its virtue lies in elucidating the fundamental characteristics of elastic wave behavior in locally resonant systems through explicit formulations, unobscured by the complexity of the problem. Furthermore, the outcomes derived from this less restrictive model possess broader universality, proving applicable to complex extended models, thereby reinforcing the robustness and versatility of our findings.


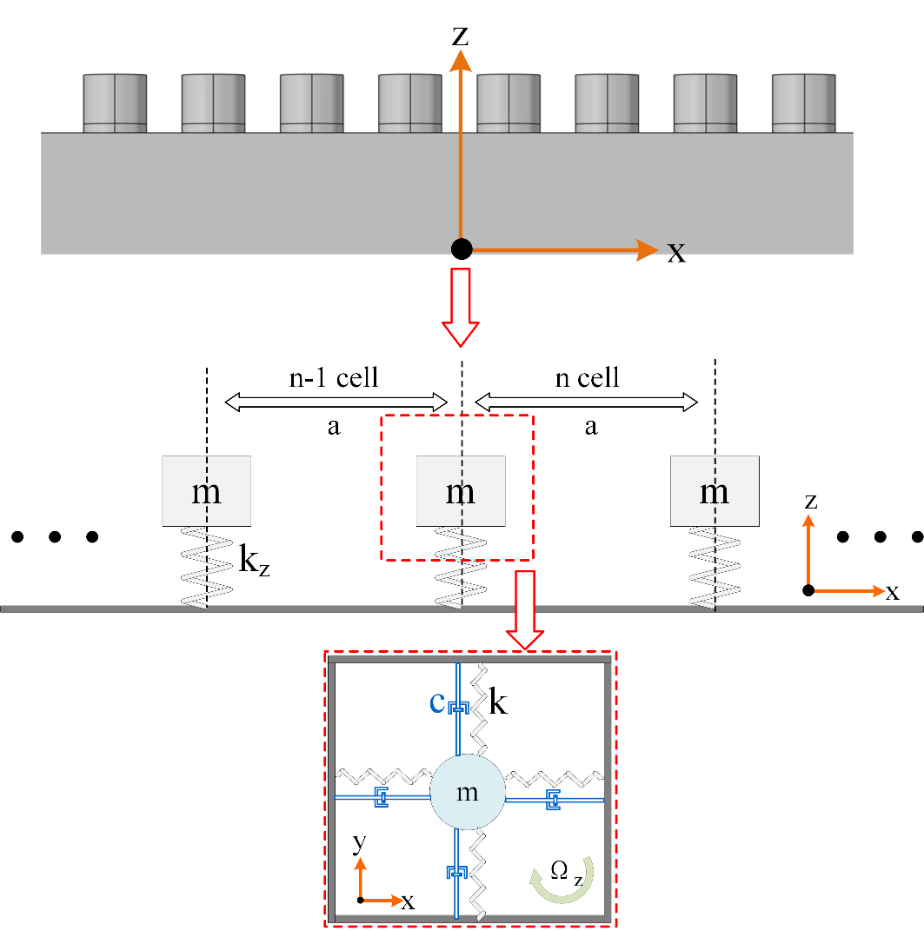


**Fig.1 Equivalent model of PM under rotation.** WGMs of the hollow pillar is equivalent to a system of periodically distributed spring-mass elements on a string, and the mass elements experience rotational coupling in the x-y plane.

**Frequency splitting under rotation.**

The WGM1 and WGM2 are a pair of degenerate modes, sharing the same resonant frequency and vibrational characteristics but exhibiting orthogonal vibration directions in the x-y plane. Therefore, their respective displacements within the mass-spring system can be represented by [*x, z_x_*] and [*y, z_y_*], with the displacement of each mass element being the vector linear superposition of these two components [*x, y, z=z_x_+z_y_*]. When accounting for the rotation effects, since the Coriolis effect act solely within the x-y plane (dictated by the degeneracy of the vibration), the rotational coupling equations for the spring-mass system, in accordance with Eq.1 presented in the manuscript, can be formulated as follows:

(1)

The equations can be solved by the rotating-wave approximation method. Assuming the solutions for displacements x and y are given by:

(2)

where and are the slowly varying complex amplitudes, and the asterisk superscript denotes the conjugate transformation. Substituting Eq.2 into Eq.1 and neglecting the second derivative terms (slightly varying terms) and centrifugal force components (rotation rate is much lower than the resonance frequency), we obtain:

(3)

$\mathbf{H}=\left[\begin{matrix} \frac{\omega_{0}^{2}-\omega^{2}}{2\omega}+\frac{ic}{2m} & \frac{im_{cori}\Omega_{z}}{m} \\ \frac{-im_{cori}\Omega_{z}}{m} & \frac{\omega_{0}^{2}-\omega^{2}}{2\omega}+\frac{ic}{2m} \end{matrix}\right]$ (4)

where $\omega_{0}=\sqrt{k/m}$ represents the characteristic frequencies of the system without rotational coupling, while ***H*** denotes the dynamic matrix, whose real parts of the eigenvalues yield the characteristic frequencies of the system under the influence of rotational coupling. Ultimately, this leads us to the formulation encapsulated in Manuscript Eq.2 ($\omega=\omega_{0}\pm m_{cori}\Omega_{z}/m$). In alignment with the characteristics of the model presented in the manuscript, we set the initial resonant frequency $f_{0}=\omega_{0}/2\pi$ at 38.7 MHz, effective mass $m=2.64e^{-8}kg$, Coriolis mass $m_{cori}=1.56e^{-8}kg$ and the quality factor $Q=1935$ ($Q=\omega_{0}m/c=f_{0}/\left|f_{WGM1}-f_{WGM2}\right|$). And trends of the resonant frequency shifts under growing rotation are plotted as Fig.2a.

To illustrate this conclusion concretely, considering a case of forced vibration along a single direction, the rotational coupling equations for the open-loop system are recast as follows:

(5)

Substituting Eq.2 into Eq.5, we attain the following analytical solution:

$\left\{\begin{matrix} \left|x\right|=\sqrt{\frac{(\omega_{0}^{2}-\omega_{drive}^{2})^{2}+c^{2}\omega_{drive}^{2}/m^{2}}{[(\omega_{0}^{2}-\omega_{drive}^{2})^{2}-c^{2}\omega_{drive}^{2}/m^{2}-4m_{cori}^{2}\Omega_{z}^{2}\omega_{drive}^{2}/m^{2}]^{2}+4(\omega_{0}^{2}-\omega_{drive}^{2})^{2}c^{2}\omega_{drive}^{2}/m^{2}]}} \\ \left|y\right|=\sqrt{\frac{4m_{cori}^{2}\Omega_{z}^{2}\omega_{drive}^{2}/m^{2}}{[(\omega_{0}^{2}-\omega_{drive}^{2})^{2}-c^{2}\omega_{drive}^{2}/m^{2}-4m_{cori}^{2}\Omega_{z}^{2}\omega_{drive}^{2}/m^{2}]^{2}+4(\omega_{0}^{2}-\omega_{drive}^{2})^{2}c^{2}\omega_{drive}^{2}/m^{2}]}} \end{matrix}\right.$ (6)

Under forced vibration, the displacement responses of the rotational coupling spring-mass system are depicted as Fig.2b and Fig.2c. Figure 2b and 2c, in addition to exhibiting the frequency splitting phenomenon consistent with Fig.2a, further reveals that as the angular velocity increases, the amplitude in the x-direction gradually decreases and stabilizes, whereas the displacement in the y-direction grows from zero to a steady value. This illustrates the Coriolis effect's role in redistributing energy between the two modes.

**Attenuation characteristics of the PM.** We now turn our attention to considering the attenuation characteristics of elastic waves by such periodically arranged local resonance units, along with the implications of rotation on these properties. Assuming the tension (elastic constant) in the string is denoted by T and the mass density by ρ, the wave equation governing the propagation of elastic waves along the string is satisfied as follows:

$\frac{\partial^{2}u}{\partial t^{2}}=v^{2}\frac{\partial^{2}u}{\partial x^{2}}$ (7)

where 𝑢 represents the displacement of the string in the z-direction, and $v=\sqrt{T/\rho}$ denotes the propagation speed of the elastic wave along the string. With the periodic resonators partitioning the string into numerous segments, the general solution for the displacement within the n-th unit of the string can be expressed as:

(8)

where $x_{n}=x-na$, *a* represents the periodic constant between resonators, *C_n_* and *D_n_* are coefficients to be determined, $q=\omega/v$ is the wave number of the string without attachments. For the n-th resonator, since there is no coupling between the displacements of the two degenerate modes along the z-direction, the vibrations of the two degenerate modes along the z-direction satisfy the dynamic equations separately:

$m\frac{\partial^{2}Z_{nx/ny}(t)}{\partial t^{2}}+k_{zx/zy}[Z_{nx/ny}(t)-u(x_{n}|_{x_{n}=0},t)]=0$ (9)

where $Z_{nx}(t)=z_{nx}e^{i\omega t}$ and $Z_{ny}(t)=z_{ny}e^{i\omega t}$ represent the displacements of the two degenerate modes along the z-direction for the n-th resonator, respectively. *k_zx_* and *k_zy_* are the equivalent spring constants in the z-direction for the two degenerate modes, varying with the rotation. Substituting Eq.8 and 9 into Eq.7 yields the relationship between the displacement of the elastic wave at the location of the n-th resonator and the displacement of the two degenerate modes along the z-direction as follows:

$Z_{nx/ny}(t)=\frac{k_{zx/zy}D_{n}}{k_{zx/zy}-m\omega^{2}}e^{i\omega t}$ (10)

Thus, the interaction force between the string and the n-th resonator can be expressed as:

$\begin{matrix} F_{n}=k_{zx}[z_{nx}-u(x_{n}|_{x_{n}=0},t|_{t=0})]+k_{zy}[z_{ny}-u(x_{n}|_{x_{n}=0},t|_{t=0})] \\ =[\frac{k_{zx}m\omega^{2}}{Tq(k_{zx}-m\omega^{2})}+\frac{k_{zy}m\omega^{2}}{Tq(k_{zy}-m\omega^{2})}]D_{n} \end{matrix}$ (11)

At the interface between the (n-1)-th and n-th unit of the string, the continuity conditions for displacement and stress must be satisfied:

$\left\{\begin{matrix} u(x_{n-1}|_{x_{n-1}=a},t|_{t=0})=u(x_{n}|_{x_{n}=0},t|_{t=0}) \\ T\frac{\partial u(x_{n},t)}{\partial x}|_{x_{n}=0,t=0}-Fn=T\frac{\partial u(x_{n-1},t)}{\partial x}|_{x_{n-1}=a,t=0} \end{matrix}\right.$ (12)

And this leads to the transfer expressions between adjacent units:

$\begin{matrix} \left[\begin{matrix} C_{n} \\ D_{n} \end{matrix}\right]=\left[\begin{matrix} -P\sin(qa)+\cos(qa) & -P\cos(qa)-\sin(qa) \\ \sin(qa) & \cos(qa) \end{matrix}\right]\left[\begin{matrix} C_{n-1} \\ D_{n-1} \end{matrix}\right] \\ =\mathbf{T}\left[\begin{matrix} C_{n-1} \\ D_{n-1} \end{matrix}\right] \end{matrix}$ (13)

where ***T*** is the transfer matrix between adjacent units, and $P=F_{n}/D_{n}$. Additionally, due to the periodic arrangement of the resonators, the Bloch periodicity condition must be satisfied between adjacent units:

$\left[\begin{matrix} C_{n} \\ D_{n} \end{matrix}\right]=e^{i\beta a}\left[\begin{matrix} C_{n-1} \\ D_{n-1} \end{matrix}\right]$ (14)

where $\beta$ is the Bloch wave vector along the string. Combining Eq.14 and 15, we obtain the eigen matrix for the locally resonant system:

$\left|\mathbf{T}-e^{i\beta a}\mathbf{I}\right|=0$ (15)

where ***I*** is the unit matrix, and the dispersion relation of $\beta$ with angular frequency $\omega$ can be obtained by:

$\cos(\beta a)=\cos(qa)-P\sin(qa)/2$ (16)

where the imaginary part of $\beta a$ is referred to as the attenuation constant, which characterizes the attenuation properties of the elastic wave propagation. Based on the characteristics of the model presented in the manuscript, we set the mass density of the string $\rho$ to 2329 kg/m^3^ and the elastic wave velocity $v$ to 5355 m/s. Figure 2d plots the attenuation constant of elastic waves under different angular velocity. It can be seen that the elastic waves experience attenuation at the localized resonance mode, and as the angular velocity increases, the attenuation peak splits linearly.


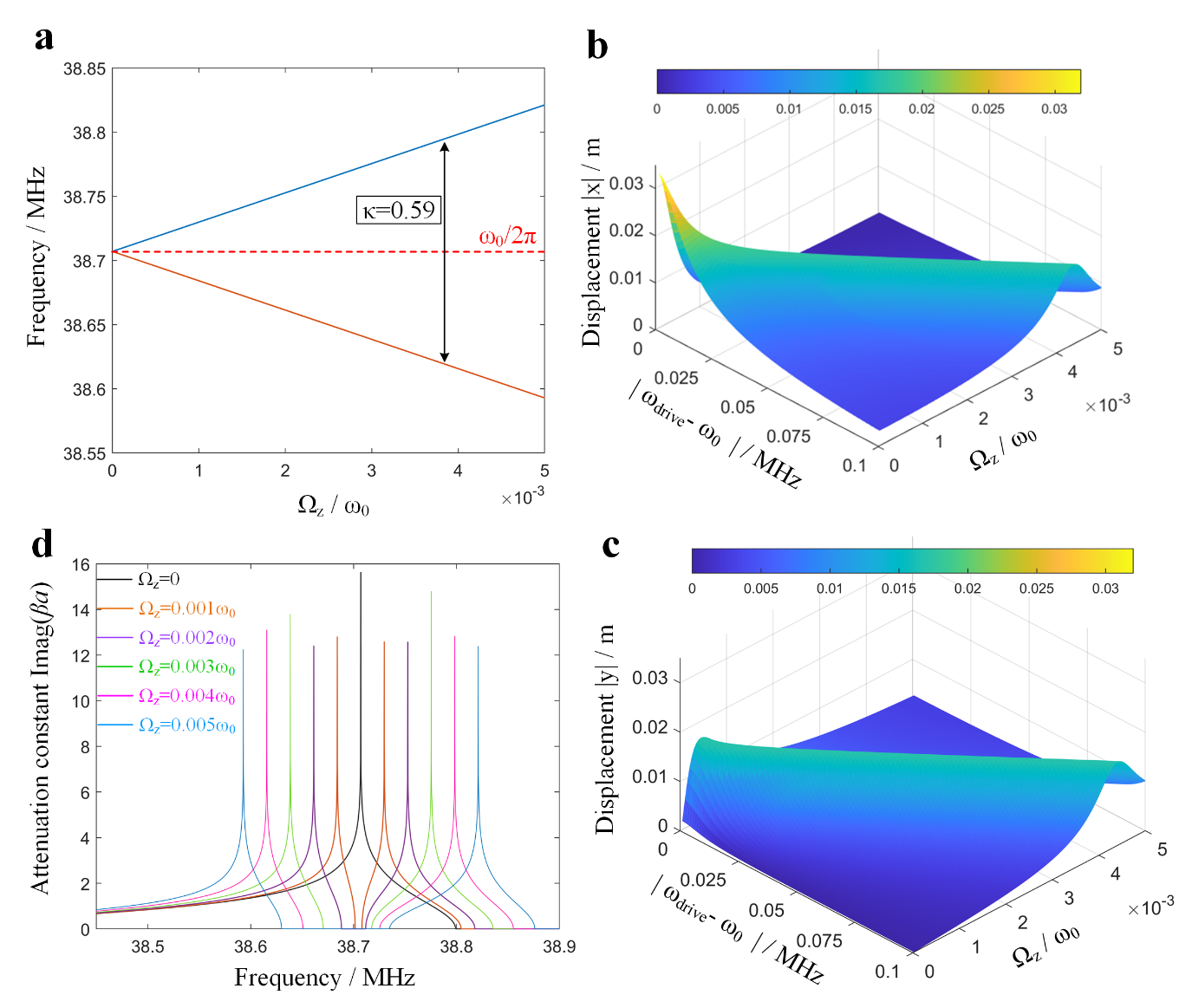


**Fig.2 Results of the equivalent model. a.** Trends of the resonant frequency shifts under growing rotation. **b.** Displacement response in x-direction under forced vibration. **c.** Displacement response in y-direction under forced vibration. **d.** Attenuation characteristics of the PM under growing rotation, obtained by the TMM.
