# Supplementary figures and images for: A novel gyroscope based on the slow surface acoustic wave in a phononic metamaterial
Source: Microsyst Nanoeng. 2024 Nov 14;10:169. doi: 10.1038/s41378-024-00787-1 (PMC11564662; doi:10.1038/s41378-024-00787-1)

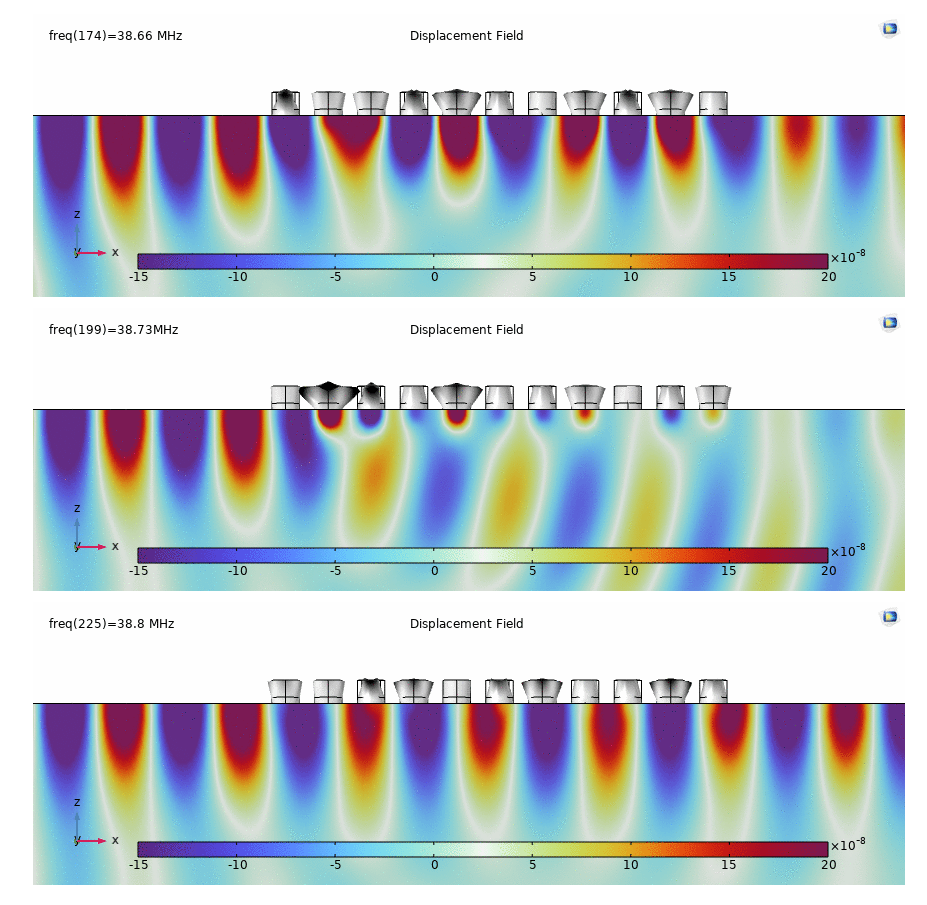

Supplement: Supplementary file 3 — supplementray_video1_varying_phase_velocity [file 41378_2024_787_MOESM3_ESM.gif]

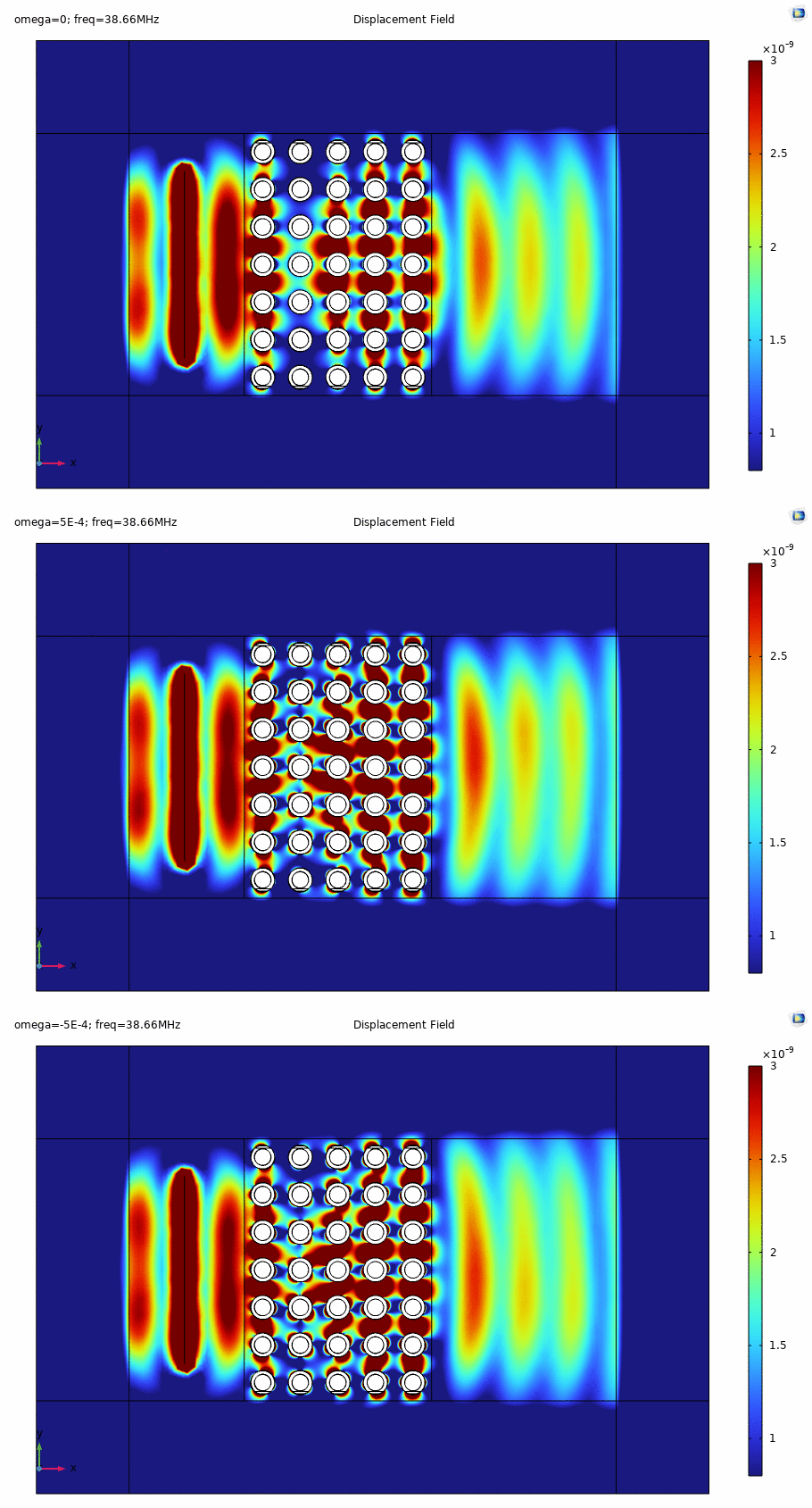

Supplement: Supplementary file 4 — supplementray_video2_rotation_direction [file 41378_2024_787_MOESM4_ESM.gif]
